# Respiratory muscle function in the newborn: a narrative review

**DOI:** 10.1038/s41390-021-01529-z

**Published:** 2021-04-19

**Authors:** Theodore Dassios, Aggeliki Vervenioti, Gabriel Dimitriou

**Affiliations:** 1 Department of Women and Children’s Health, King’s College London, London, UK; 2 Department of Paediatrics, University of Patras, Patras, Greece

## Abstract

**Abstract:**

Our aim was to summarise the current evidence and methods used to assess respiratory muscle function in the newborn, focusing on current and future potential clinical applications. The respiratory muscles undertake the work of breathing and consist mainly of the diaphragm, which in the newborn is prone to dysfunction due to lower muscle mass, flattened shape and decreased content of fatigue-resistant muscle fibres. Premature infants are prone to diaphragmatic dysfunction due to limited reserves and limited capacity to generate force and avoid fatigue. Methods to assess the respiratory muscles in the newborn include electromyography, maximal respiratory pressures, assessment for thoraco-abdominal asynchrony and composite indices, such as the pressure–time product and the tension time index. Recently, there has been significant interest and a growing body of research in assessing respiratory muscle function using bedside ultrasonography. Neurally adjusted ventilator assist is a novel ventilation mode, where the level of the respiratory support is determined by the diaphragmatic electrical activity. Prolonged mechanical ventilation, hypercapnia and hypoxia, congenital anomalies and systemic or respiratory infection can negatively impact respiratory muscle function in the newborn, while caffeine and synchronised or volume-targeted ventilation have a positive effect on respiratory muscle function compared to conventional, non-triggered or pressure-limited ventilation, respectively.

**Impact:**

- Respiratory muscle function is impaired in prematurely born neonates and infants with congenital anomalies, such as congenital diaphragmatic hernia.
- Respiratory muscle function is negatively affected by prolonged ventilation and infection and positively affected by caffeine and synchronised compared to non-synchronised ventilation modes.
- Point-of-care diaphragmatic ultrasound and neurally adjusted ventilator assist are recent diagnostic and therapeutic technological developments with significant clinical applicability.

## Introduction

The main muscle of inspiration is the diaphragm, which is a thin, dome-shaped skeletal muscle that contracts during inspiration and generates negative intra-thoracic pressure that draws air in the thoracic cavity. Other muscles that contribute to inspiration are the intercostal muscles, the sternocleidomastoids and the scalenes, which assist in elevating the rib cage.^1,2^ The clinical importance of the respiratory muscles lies in that they undertake the work of breathing (WoB), and when their functional integrity is compromised, independent breathing cannot be sustained, resulting in a need for assisted ventilation. An estimation of the predicted ability of the respiratory muscles to independently undertake the WoB is used in the assessment of readiness for extubation from mechanical ventilation.^3^ Our aim was to review the currently available evidence on respiratory muscle function in the newborn and describe the methods used to assess respiratory muscle function in this population, focusing on current and future potential clinical applications.

We searched PubMed, Scopus and the Cochrane Central trials register up to November 2020 and performed a manual search of references in narrative and systematic reviews. Search terms included “infant”, “newborn”, “respiratory muscles”, “work of breathing”, “diaphragm” and “diaphragmatic”. No predefined age cut-off was used in the definition of a “newborn”, as extremely preterm infants measured at term would fall outside the epidemiological definition cut-off of 28 days of age.”

### The diaphragm prenatally and at birth

Foetal breathing movements are essential for the intrauterine development of the respiratory muscles. In the healthy human foetus, breathing activity can be detected from 10 to 12 weeks of gestation and occurs at an average frequency of 60 per minute.^4^ Seminal in utero animal studies that analysed the contractile and fatigue properties of the diaphragm demonstrated that during intrauterine life twitch contraction and relaxation times decrease, tetanic force levels increase and the range of forces generated by the diaphragm in response to graded nerve stimulation increases.^5^ These changes functionally allow the diaphragm to regulate the movement of the very compliant rib cage in the newborn.^5^ At birth, the contraction of the diaphragm develops an oesophageal pressure swing up to −70 cm H_2_O^6^ and the newborn closes the glottis to maintain a positive intra-thoracic pressure and facilitate air distribution inside the lungs.^7^ A study of eight infants born between 28 and 31 gestational weeks and supported by early nasal continuous positive airway pressure (nCPAP) reported that the median electrical activity of the diaphragm peak value was 19.2 μV at 20 min after birth, which decreased to 11.4 μV at 55 min of age.^8^ The same study highlighted that preterm infants were capable of generating sufficient diaphragm tone during expiration to establish and maintain functional residual capacity (FRC). Diaphragm activity decreased during the first 90 min, suggesting that early adaptation was accomplished by 90 min of age.^8^

## The neonatal respiratory pump: anatomical and mechanical considerations

Although in adults elevation of the ribs during inspiration increases the intra-thoracic volume, the newborn ribs are already elevated and horizontal, and their elevation produces little effect on the intra-thoracic volume.^9^ Furthermore, the non-ossified, highly compliant neonatal chest wall^10^ renders the neonatal thorax prone to distortion, especially when intercostal muscle activity is inhibited during rapid eye movement (REM) sleep.^11^ Unlike the adult dome-shaped diaphragm, the neonatal diaphragm is morphologically flattened and inserted to the chest wall with a larger angle, resulting in a smaller zone of apposition and decreased range of displacement.^12^ In adults, the zone of apposition serves as a reserve from which the diaphragm contracts caudally like a piston, which results in inward air flow.^13^ In the newborn, however, the diaphragm acts more as a bellow, its posterior limb moving dorsally and when in respiratory distress can distort the lower part of the rib cage in what is clinically described as “subcostal recession”. Structurally, the newborn diaphragm consists of fewer fatigue-resistant slow twitch (type-I) fibres, decreased oxidative capacity and low total cross-sectional area of all fibre types.^14^ The poor functional reserve of the newborn diaphragm renders it prone to muscle fatigue. When the WoB is increased, the accessory muscles (sternocleidomastoid and scalene) are recruited in order to avoid fatigue, coupled with post-inspiratory diaphragm contraction and laryngeal braking, a phenomenon that manifests clinically as expiratory “grunting”.^15^ Post inspiratory diaphragm activation coupled with upper airway muscle activation are also present in non-laboured tidal breathing in term infants in an effort to control airflow throughout the breathing cycle.^16^ Post inspiratory diaphragm activation ensures a controlled expiration to prevent the newly recruited alveoli from collapsing and results in increased FRC and improved gas exchange,^17^ while in laboured breathing, expiration in preterm neonates becomes an active phenomenon, during which the abdominal muscles are activated.^18^

## Assessment of respiratory muscle function

### Electromyography (EMG)

Surface diaphragmatic EMG was historically the first method applied in the neonatal population to assess diaphragmatic physiology.^19^ The initial studies described that surface diaphragmatic EMG in fatigue is characterised by a fall in high frequencies (160–640 Hz) and a rise in low frequencies (10–40 Hz) and that a shift towards the low frequencies indicates fatigue.^20^ When the diaphragm fatigues, the intercostal muscles act synergistically to counteract the impaired diaphragmatic performance^21^ and to stabilise the neonatal thorax.^22^ Sustained airway occlusion results in increased surface EMG peak amplitude in unsedated newborn preterm infants.^23^ REM sleep is associated with loss of intercostal muscle tone in neonates.^11,24^ Carbon dioxide rebreathing in healthy preterm infants causes increased EMG activity as an adaptive diaphragmatic post-inspiratory response to maintain end-expiratory lung volume.^25^

While studying the EMG responses of the upper-airway muscles and the diaphragm to inspiratory resistive loading, the diaphragm increases its EMG activity in response to induced flow restriction.^26^ Another study described that, during obstructive apnoea, premature infants respond by increasing the EMG activity of the diaphragm and decreasing the EMG activity of the upper-airway muscles,^27^ probably in an effort to accommodate more inward airflow. Hutten et al. described that the EMG activity of the intercostal muscles and the diaphragm is related to the resistive load of breathing, externally imposed by a face mask,^28^ and premature infants with chronic lung disease activate their inspiratory muscles earlier during expiration than healthy term infants.^29^

### Measurement of respiratory pressures and composite indices

In infants, maximal static inspiratory (*P*_Imax_) and expiratory (*P*_Emax_) pressures are recorded as the most negative and most positive pressures, respectively, generated during crying against an occluded airway and can be measured at the level of the mouth.^6^ Crying is assumed to represent a maximal effort^30^ and *P*_Imax_ and *P*_Emax_ are measured starting from a volume approaching the residual volume and total lung capacity, respectively.^31,32^ Mean maximal transdiaphragmatic pressure has been reported to be 72 cm H_2_0 in term infants measured at a mean age of 11.6 months post conception and correlates with postconceptional age, reflecting a developmental/maturation effect.^33^ Mean *P*_Imax_ during crying in healthy term infants measured later at a mean age of 1.4 years was 118 cm H_2_O and was independent of age, sex and anthropometry, while mean *P*_Emax_ was 125 cm H_2_O and correlated with body weight.^31^ In premature infants, maximal airway pressures during crying were significantly lower: the major determinant of *P*_Imax_ was postconceptional age and of *P*_Emax_, body weight at study.^34^ Smaller and lighter infants failed extubation more frequently, but *P*_Imax_^35,36^ or maximal transdiaphragmatic pressure during crying^37^ did not independently predict extubation outcome. It should be noted that assessment of respiratory muscle function by maximal static pressures is indirect and effort-dependent; low values are not always indicative of respiratory muscle weakness and the scatter of normal values is relatively large.^38^

The tension time index (TTI) of the diaphragm or of the aggregate of all the inspiratory muscles is the product of the ratio of the mean inspiratory pressure (defined primarily by the occlusion pressure) to the *P*_Imax_ times the ratio of the inspiratory time to the total breathing cycle time.^39^ In concept, the longer the inspiratory muscles contract during a breathing cycle and the higher fraction of their maximum force they use, the less efficient and more fatigue-prone they are. Higher TTI values signal inefficient inspiratory muscle function and increased risk of respiratory muscle fatigue under conditions of increased respiratory load. This index can either be measured based on the transdiaphragmatic pressures measured via an indwelling oesophageal catheter or non-invasively with pressure measurements at the level of the mouth.^36,40^ As highlighted later in this review, both techniques have been used to predict extubation outcome, as ventilated infants who eventually fail extubation produce higher TTI values.^36,40^

### Phrenic nerve stimulation

Although not part of routine clinical care, direct and non-volitional assessment of diaphragmatic function can be performed by phrenic nerve stimulation and consequent measurement of the phrenic nerve latency times,^41^ the evoked diaphragm EMG^32^ and the transdiaphragmatic pressure.^42^ In a study that included neonates with a median gestational age of 35 weeks, electrical and magnetic stimulation of the phrenic nerve was used to assess neonatal diaphragmatic function in a non-volitional manner.^43^ Transdiaphragmatic pressure values generated by magnetic stimulation of the phrenic nerves are related to gestational age at birth and postconceptional age at study.^44^ Infants with abdominal wall defects and congenital diaphragmatic hernia (CDH) exhibit a trend for prolonged latency times and increased activation of the diaphragmatic EMG potential.^45^ It should be noted, however, that technical difficulties might preclude the application of magnetic stimulation in very small or very premature infants as the relatively large magnetic coil would be difficult to apply in the small neck of a premature infant. Furthermore, electrical nerve stimulation either via a needle or via an indwelling wire is painful and might not be tolerated well in this population. These difficulties might preclude the application of this method in the clinical setting in the near future, but it would be interesting, if the technical difficulties were addressed (for example if a smaller magnetic coil was to become available), to use this technique to study how diaphragmatic function is affected in conditions such as prolonged mechanical ventilation or in the presence of systemic or respiratory infection (Table 1).

**Table 1:** Methods to assess respiratory muscle function and the work of breathing.

	Methodology	Strengths and clinical relevance	Prediction of extubation, highest reported area under the curve	Limitations
Electromyography (EMG)	Detection of the electrical signal of the diaphragm on the surface or with indwelling oesophageal and gastric catheters	Sensitive detection of the start of a breathing effort and provides insight in the amount of breathing effort, applicable in NAVA ventilation	0.77^60^	Specialised equipment is required
Maximal respiratory pressures (*P*_Imax_, *P*_Emax_)	Measurement of the maximal pressures during inspiration and expiration generated during crying against an occluded airway	Maximal pressures are a surrogate for muscle strength and increase with maturation	0.90^35^	Assessment is effort-dependent. Large scatter of normal values
Phrenic nerve stimulation	Electric or magnetic stimulation of the phrenic nerve and measurement of the diaphragm EMG and transdiaphragmatic pressure	Non-volitional method	Has not been assessed in neonates	Specialised equipment is required
Tension Time Index (TTI) of the diaphragm	Product of the ratio of the mean transdiaphragmatic pressure to the maximum inspiratory transdiaphragmatic pressure times the ratio of the inspiratory time to the total breathing cycle time	Composite index of respiratory muscle efficiency: less efficient function when inspiration involves a high proportion of the maximal inspiratory pressure and happens during a large part of the respiratory cycle	1.00^40^	Specialised equipment and post measurement analysis are required
Thoraco-abdominal asynchrony (TAA)	Lack of synchrony between the chest and abdomen during respiration and calculation of the corresponding phase angle	Non-invasive method. Continuous positive airway pressure decreases TAA. Asynchrony decreases post feeding	Not applicable	Only useful in non-ventilated infants
Relaxation rate of the respiratory muscles	A longer time to relax after contraction signals respiratory muscle fatigue	Ventilator pressure waveforms can be used as a surrogate for calculating the rate of relaxation. Less efficient respiratory muscle function in the presence of systemic infection and in infants of a lower gestational age	0.937^53^	Difficult calculations. Currently not available in real time
Diaphragmatic ultrasound	The thickness of the diaphragm and the range of diaphragmatic displacement can be non-invasively measured	Inexpensive, non-ionising and accessible method. Diaphragmatic velocity decreased during fatigue. Diaphragmatic thickness is higher in term compared to preterm infants	0.98^63^	More meaningful in spontaneous breathing infants

### Thoraco-abdominal asynchrony (TAA) and WoB

**Fig. 1:**
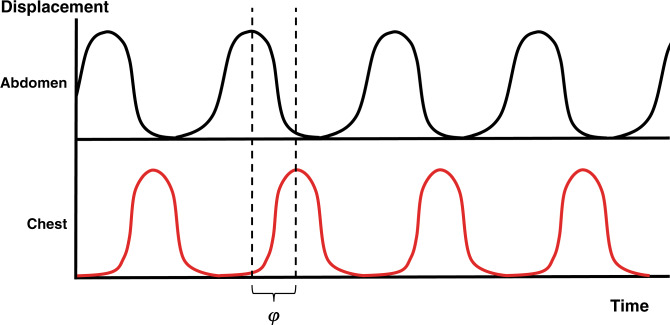
Chest and abdomen displacement over time. When the two compartments move in asynchrony (thoraco-abdominal asymmetry), the phase angle (*φ*) between the movement of the two compartments can be calculated.

While in healthy individuals there is synchronous inward and outward motion of the chest and abdomen, non-synchronous movement of the two compartments occurs in TAA. The asynchrony can be measured by respiratory inductive plethysmography and quantified by the phase angle between the chest and abdominal movement, when the relevant displacements are synchronously plotted against time (Fig. 1).^46^ TAA and the corresponding phase angle are not strictly indices of respiratory muscle function but can be used to assess the WoB.^32^ Infants with bronchopulmonary dysplasia (BPD) exhibit marked TAA,^47^ while CPAP decreases TAA.^48^ When compared to healthy infants, children who were diagnosed with severe BPD demonstrate a different pattern of TAA in toddler age consisting of increased thoracic inspiratory efforts and expiratory muscle activity.^49^ Unlike infants without respiratory problems, mechanically ventilated infants or infants in respiratory distress demonstrate pronounced abdominal muscle activity during expiration.^18^ Furthermore, in response to CO_2_ rebreathing, the abdominal muscles are recruited in healthy preterm neonates.^50^ The degree of TAA and the corresponding phase angle is significantly decreased in the immediate post-feeding period in convalescent healthy neonates compared to the period immediately before feeding.^51^

### Relaxation rate of the respiratory muscles

When skeletal muscles contract against increased external loads, their relaxation slows and fatigued muscle fibres take longer to relax after contraction.^52^ The rate of relaxation can be measured by plotting the proximal airway pressure change over time.^32^ In 46 ventilated infants with a median gestation of 26 weeks, a prolonged relaxation phase during spontaneous breathing was used to accurately predict extubation failure independently of gestational age or age at study.^53^

### Diaphragmatic ultrasound

**Fig. 2:**
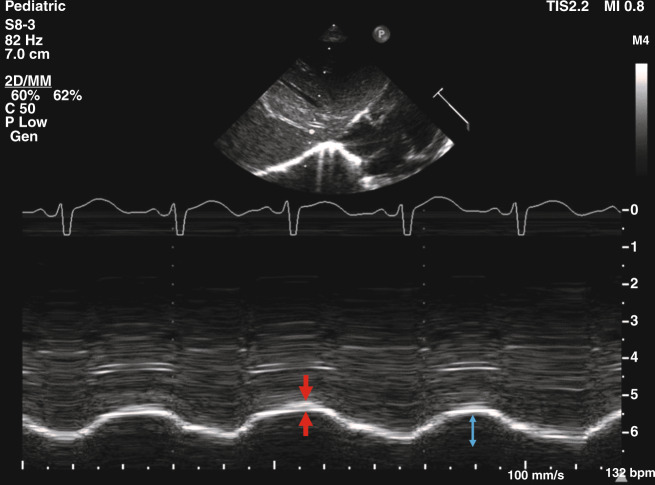
Diaphragmatic ultrasound Sonographic image showing M-mode measurement of diaphragmatic thickness (between the two arrows) and excursion of the right hemidiaphragm (bidirectional arrow) in a healthy term infant.

Point-of-care diaphragmatic ultrasound is emerging as a popular bedside clinical tool in neonatal intensive care.^54^ Ultrasonographic assessment of the diaphragm can be undertaken from the right subcostal area, using the liver acoustic window to measure diaphragmatic thickness in the zone of apposition as well as diaphragmatic kinetics by M-mode ultrasonography and recording the displacement of the diaphragmatic segments and velocity of motion (Fig. 2)^55–57^ Normative data have been established in healthy term infants.^56^ While diaphragmatic motion in healthy infants occurs predominantly in the posterior segment, this predominance is lost in mechanically ventilated and muscle-relaxed infants.^56^ Following hypercapnia and induced fatigue by phrenic nerve pacing in piglets, the inspiratory velocity of the diaphragm decreased significantly, finding that reversed during recovery.^57^ The median diaphragmatic thickness was higher in 33 term compared to 33 preterm infants highlighting increasing skeletal muscle mass with increasing maturity but the diaphragmatic shortening fraction was not different between the two groups.^58^

### Ventilator waveforms

Although the ventilator waveforms are not methods to specifically assess respiratory muscle function, some valuable information can be indirectly obtained by observing these graphics at the cot side. Asynchrony between the patient and the ventilator can be observed in the flow versus time waveform as delayed triggering, ineffective inspiratory effort that fails to trigger a mechanical breath or as presence of two or more mechanical breaths delivered during a single respiratory effort.^59^ A prolonged time taken by the pressure waveform to return to baseline following a spontaneous breathing effort describes impaired respiratory muscle function and impeding or established muscle fatigue.^53^

## Respiratory muscle indices to predict readiness for extubation and weaning from non-invasive support

Although respiratory muscle indices are not the only predictors of extubation outcome, impaired respiratory muscle function pre-extubation has been assessed as a possible predictor of extubation failure, especially in premature infants that often fail to successfully wean off invasive support.^60^ Eighteen ventilator-dependent preterm infants with respiratory distress syndrome were studied for 24 h before and after an attempt at extubation. The infants that were successfully extubated had a greater maximal inspiratory force before extubation compared to the ones that failed extubation.^61^ Dimitriou et al. measured the maximum inspiratory pressure during airway occlusion in 36 infants with a median gestational age of 31 weeks and reported that the 7 infants who failed extubation generated lower maximum inspiratory pressures than the ones who succeeded, but gestational and postnatal age were better predictors of extubation failure.^35^ Currie et al. measured the TTI in 20 ventilated infants, with a median gestational age of 31 weeks prior to extubation and reported that the five infants that failed extubation had significantly higher values than the successfully extubated infants.^36^ Bhat et al. in a follow-on study of the same group with 60 infants and a median gestational age of 35 weeks reported that both the invasive diaphragmatic and the non-invasive respiratory muscle TTI were higher in infants who failed extubation but did not perform significantly better in predicting the outcome of extubation than gestational age or birth weight.^62^ Another study, though, by Dimitriou et al. of 56 preterm infants reported that the TTI of the diaphragm had zero false-positive or false-negative results in predicting extubation outcome.^40^ These differences could be explained by population characteristics as the study of Bhat et al. included more mature infants with a median gestation of 35 weeks compared to 30 weeks in Dimitriou et al. It is plausible that the reason for extubation failure in more mature infants could be less related to immaturity and impaired muscle function and more to other comorbidities, such as infection or congenital anomalies.

A study of 46 mechanically ventilated infants with a median gestational age of 26 weeks reported that a respiratory muscle time constant of relaxation that increased >1.02 s/cm H_2_O during spontaneous breathing predicted extubation failure with 94% sensitivity and 83% specificity.^53^ In a group of 43 ventilated infants <32 weeks of gestation that underwent sonographic evaluation of the diaphragmatic thickness and excursion as a predictor for successful extubation, the infants who succeeded had a significantly higher diaphragmatic thickness and excursion, compared to infants who failed extubation.^63^

Respiratory muscle assessment can also be used in titrating non-invasive respiratory support during weaning. In a group of 59 stable preterm infants with a mean gestational age of 29 weeks, weaning from nasal CPAP to low-flow nasal cannula at 1 l/min was associated with an increase in the EMG amplitude and infants failing the weaning attempt tended to have a higher diaphragmatic activity than the ones who were successfully weaned.^64^ In another study of 18 infants of a median gestation of 27.8 weeks measured at a median postnatal age of 54 days, the mean amplitude of the EMG was not different at high flow rates of 4, 6 and 8 l/min.^65^

## Factors that impact on respiratory muscle function

Respiratory muscle function is influenced by epidemiological factors such as gestation age and weight, as described earlier in this review.^31,33,44^ In REM sleep, intercostal muscle tone was lost^66^ and post-inspiratory diaphragmatic activity was reduced.^67^ Thus, the expiratory flow braking mechanisms were disabled leading to a relative loss of FRC. On the contrary, hyperinflation and increased FRC, in the context of meconium aspiration syndrome or ventilator-induced overexpansion, could place the respiratory muscles at a mechanical disadvantage, as marked hyperinflation would force the diaphragm to flatten and displace caudally.^68^ This lead to a decreased zone of apposition and decreased force-generating capacity based on the force–length relationship of the diaphragm, where the optimal length at which the greatest force output of the diaphragm was generated, corresponded to a lung volume marginally lower than the FRC.^69^ Finally, infants with chronic lung disease, in an effort to counteract distorted lung mechanics and decreased FRC, maintained normal lung volumes by starting to inspire earlier in the respiratory cycle.^29^

### Gas exchange

Respiratory muscle dysfunction, evidently, can be the cause of hypercapnia,^70^ but hypercapnia can also impact respiratory muscle function. Piglet experiments have demonstrated that severe hypercapnia has a depressant effect on diaphragmatic force output^71^ and acute hypoxaemia impaired the capacity of the developing diaphragm to generate force, with young piglets being more resistant to the depressant effects of hypoxaemia, when compared to their older counterparts.^72^ Hypercapnia and diaphragmatic fatigue had a synergistic negative effect on diaphragmatic function, as assessed by ultrasonography.^57^ In seven adult humans, hypercapnia did not intensify long lasting fatigue but reduced the diaphragm contractility, which was assessed by measurement of the maximal inspiratory pressure.^73^

### Posture

Prone position has been shown to decrease the degree of TAA, compared to supine.^74^ Prone position was associated with decreased TAA and WoB in non-ventilated premature infants.^75^ However, *P*_Imax_ was higher in the supine position in late preterm infants, probably due to increased lung volume and greater diaphragm shortening in the prone position.^76^ Occlusion pressure and *P*_Imax_ were lower in the prone, compared to the supine position.^77^ Convalescent premature infants loaded with CO_2_ and studied prone and supine demonstrated a higher FRC and occlusion pressure in the supine position compared to prone.^78^ Finally, the effect of posture on oxygenation and on the WoB, assessed by the measurement of the diaphragmatic pressure–time product (PTP), in convalescent preterm infants was examined. Forty infants were studied in the supine, supine with tilt of 45° and prone positions in a random order, and the WoB was lower in the prone than in the supine position and this could be a possible explanation for the higher oxygen saturation in the prone position.^79^

### Genetic factors

The I-allele of the angiotensin-converting enzyme (ACE) gene has been associated with superior muscle metabolic performance^80^ and muscle endurance.^81^ The TTI of the respiratory muscles was lower in infants with I/I ACE genotype compared to infants with one or none of the I-allele indicating an increased effectiveness of inspiratory muscles of infants with the I/I ACE genotype, due to a lower energy demand and thus to a lower risk of respiratory muscle fatigue, under conditions that increase inspiratory load.^82^

### Mechanical ventilation

Brief mechanical ventilation in rats resulted in reduced diaphragmatic weight and force-generating capacity compared to non-ventilated matched controls.^83^ Infantile diaphragms that underwent prolonged ventilation exhibited disuse atrophy, denervation atrophy and failure of normal growth and maturation.^84^ Newborn lambs that were ventilated for a mean of 8 h exhibited rapid-onset diaphragm dysfunction manifested by decreased myofibrillar force generation and increased autophagy consistent with ventilator-induced diaphragmatic dysfunction.^85^

The electrical activity of the diaphragm measured by transcutaneous EMG in 32 preterm infants with a mean gestation age of 28 weeks transitioned from nasal CPAP to high flow nasal cannula was similar in the first 3 h after transitioning.^86^ Increasing the level of CPAP from 4 to 6 cm H_2_O was associated with enhanced respiratory muscle function measured with the relaxation rate of the respiratory muscles in 38 ventilated infants with a median gestational age of 27 weeks, but there was no further benefit from increasing the pressure to >6 cm H_2_O.^87^ Compared to intermittent mandatory ventilation, patient-triggered ventilation decreased the WoB imposed on the respiratory muscles as measured by the area delimited by the curve of oesophageal pressure versus tidal volume.^88^ The PTP has been used to estimate the mechanical WoB with higher values signifying increased WoB. Comparing the PTP in CPAP and synchronised intermittent mandatory ventilation (SIMV) during weaning indicated superior efficiency of SIMV during weaning.^89^ The PTP was higher in volume-targeted ventilation targeting volumes <4 ml/kg.^90^ Targeting tidal volumes >6 ml/kg produced lower PTP (decreased WoB) compared to triggered ventilation without volume targeting.^90^ Similarly, SIMV with pressure support resulted in decreased WoB compared to SIMV without pressure support.^91^ Vervenioti et al. studied the PTP of the diaphragm in 40 preterm ventilated infants in the phase of weaning from invasive support and reported that assist control ventilation resulted in lower WoB than intermittent mandatory ventilation, while the assist control mode was further associated with a lower PTP of the diaphragm compared with SIMV.^92^ Weaning from nCPAP to low-flow nasal cannula was associated with an increase in diaphragmatic activity measured by diaphragmatic EMG and was most prominent in preterm infants failing the weaning attempt.^64^ With regards to mechanical ventilation, in conclusion, synchronised and volume-targeted modes are associated with a lower WoB compared to non-synchronised and pressure-limited modes, respectively.

### Pharmacological agents

Aminophylline increased diaphragm excursion and improved the coordination of the upper-airway muscles with the diaphragm in premature newborn infants.^93^ Caffeine increased *P*_Imax_ and *P*_Emax_ in premature neonates within 6 h of administration.^94^ Caffeine administration in 30 spontaneously breathing preterm infants with a mean gestational age of 29 weeks, resulted in a rapid (within 5 min) increase in the amplitude of transcutaneous EMG of the diaphragm, which was maintained until 120 min after caffeine administration.^95^ A loading dose of caffeine citrate given to 32 ventilated infants with a median gestational age of 29 weeks caused only a transient increase in the amplitude of the EMG peaking at 25 min post administration.^96^

Halothane anaesthesia in infants resulted in increased TAA, probably because of the effect of the agent on the intercostal muscles and the loss of their stabilising action.^97^ Administration of steroids in newborn animals resulted in diaphragm atrophy and reduction in diaphragm force, weight and endurance.^98,99^ Antenatal maternal betamethasone treatment caused postnatal diaphragmatic dysfunction at 21 days of age, which was attributed to protein loss and impairment of the anabolic signalling pathway in a rat study.^100^ Non-depolarising neuromuscular blocking agents, which are frequently used in neonatal intensive care, directly inhibited respiratory—and all other skeletal—muscle function and caused cephalad diaphragmatic displacement in response to unopposed intra-abdominal pressure.^101^

Vitamin A might play a beneficial role in neonatal diaphragmatic function. Preterm ventilated lambs that were treated with daily enteral vitamin A exhibited decreased diaphragmatic proteolysis and oxidative injury compared to placebo-treated ventilated controls.^102^

### Congenital anomalies

Infants with surgically corrected CDH and gastroschisis had lower transdiaphragmatic pressure following magnetic phrenic nerve stimulation, due to malnutrition and impaired in utero development.^42^ Furthermore, reduced phrenic nerve conduction accompanied reduced diaphragmatic strength in CDH.^45^ When seven children with repaired CDH were studied at 5 years of age, they demonstrated normal values of transdiaphragmatic pressure during crying and normal diaphragmatic TTI.^103^

### Infection

In a study of 62 ventilated newborn infants, respiratory muscle function assessed by the respiratory muscle rate of relaxation was negatively affected in the ones with previous systemic or respiratory infection, independently of gestational age at birth.^104^ In utero inflammation might also play a role. Preterm ewes delivered to mothers that received intra-amniotic injections of endotoxin before delivery exhibited impaired diaphragmatic contractility, enhanced diaphragmatic proteolysis and atrophy compared to placebo-treated controls.^105^

## Neurally adjusted ventilator assist (NAVA)

NAVA is a relatively novel mode of ventilation where the level of the delivered respiratory support is proportional to the electrical activity of the diaphragm, which is reflective of the neural respiratory drive. NAVA uses a nasogastric tube with a distal electrode array, which measures the electromyogram of the diaphragm (Edi) acting as the signal used to trigger the ventilator and determines the level of support in each inflation.^106^ When NAVA was compared to assist control ventilation in nine infants with evolving or established BPD and median gestational age of 25 weeks, NAVA improved oxygenation indices.^107^ Hunt et al. similarly compared NAVA and proportional assist ventilation in 18 infants born <32 weeks of gestation with evolving or established BPD and reported that there was no significant difference in the mean oxygenation index between the two modes, but the mean alveolar–arterial gradient was better on NAVA.^108^ NAVA can also be used as a non-invasive modality. Forty infants with a gestational age of 28–36 weeks requiring CPAP for respiratory distress were randomised to non-invasive NAVA or CPAP and the authors reported that, after 12 h of treatment, the mean FiO_2_ requirement and need for invasive ventilation did not differ between the two groups.^109^ Recently, a new non-invasive ventilation mode, which continuously adjusts the pressure support proportionally to the diaphragm electrical activity (Edi), was assessed in a cross-over feasibility study. This modality is different to NAVA in that no distinction is made between inspiration and expiration. The authors studied 20 infants of a mean gestation of 28 weeks and reported that, compared to non-invasive positive-pressure ventilation (NIPPV), the new device was well tolerated and 83% of the breaths were synchronised compared to 9% during NIPPV.^110^ Finally, transcutaneous diaphragmatic EMG was used for breath detection in spontaneous breathing preterm infants and provided similar breath detection with the Graseby capsule but with the advantage of detecting the onset of inspiration earlier.^111^

## Other interventions to alleviate the WoB

Other interventions that might alleviate the WoB by unloading the neonatal respiratory muscles include inspiratory muscle training (IMT), abdominal binding, suctioning and diuretic therapy. Successful weaning from prolonged mechanical ventilation with improved respiratory muscle strength has been reported with IMT.^112,113^ It should be mentioned, however, that these intriguing results are the product of preliminary case reports and their results should be confirmed in controlled studies before advocating respiratory muscle training in preterm neonates, which could lead to catastrophic respiratory failure. Decreasing asynchronous abdominal motion by abdominal binding decreased the WoB and stabilised the neonatal chest.^114^ Increased airway resistance would increase the afterload of the respiratory muscles; thus, airway suction and bronchodilation could theoretically decrease the respiratory muscle load. Furthermore, diuretics and chest physiotherapy could improve lung compliance and decrease the respiratory muscle afterload.^115^

## Future research considerations

Since volume-targeted ventilation and high-flow nasal therapy have become mainstream practice in neonatal clinical care, future research could focus on describing optimal settings of these modalities in reducing the WoB and facilitating transition from invasive to non-invasive support or determining readiness for transition from non-invasive support to fully unassisted breathing. The synergistic effect of impaired nutrition and steroid therapy on the WoB and the functional integrity of the diaphragm and the accessory respiratory muscles could also be investigated.

## Conclusion

Pathophysiological and anatomical factors predispose newborn infants to respiratory muscle impairment, muscle fatigue and respiratory failure. More premature infants and those with lower weight are especially prone to respiratory muscle dysfunction due to limited functional reserves. The infantile diaphragm and thorax are anatomically disadvantaged to handle an increased WoB. New interest has emerged in methods of assessing respiratory muscle function such as bedside diaphragmatic ultrasonography. Mechanical ventilation, systemic or respiratory infection, congenital anomalies and pharmacological agents can also affect respiratory muscle function.

## References

[1] Maynard, R. L. et al. *Cotes’ Lung Function* (Wiley, 2020).

[2] Dassios T (2015). Determinants of respiratory pump function in patients with cystic fibrosis. Paediatr. Respir. Rev..

[3] Shalish W, Latremouille S, Papenburg J, Sant’Anna GM (2019). Predictors of extubation readiness in preterm infants: a systematic review and meta-analysis. Arch. Dis. Child. Fetal Neonatal Ed..

[4] Natale R, Nasello-Paterson C, Connors G (1988). Patterns of fetal breathing activity in the human fetus at 24 to 28 weeks of gestation. Am. J. Obstet. Gynecol..

[5] Greer JJ (2012). Control of breathing activity in the fetus and newborn. Compr. Physiol..

[6] Gaultier C (1995). Respiratory muscle function in infants. Eur. Respir. J..

[7] LoMauro A, Aliverti A (2016). Physiology masterclass: extremes of age: newborn and infancy. Breathe (Sheff.).

[8] Oda A, Parikka V, Lehtonen L, Soukka H (2018). Rapid respiratory transition at birth as evaluated by electrical activity of the diaphragm in very preterm infants supported by nasal CPAP. Respir. Physiol. Neurobiol..

[9] Hershenson MB, Colin AA, Wohl ME, Stark AR (1990). Changes in the contribution of the rib cage to tidal breathing during infancy. Am. Rev. Respir. Dis..

[10] Papastamelos C, Panitch HB, England SE, Allen JL (1995). Developmental changes in chest wall compliance in infancy and early childhood. J. Appl. Physiol. (1985).

[11] Gaultier C, Praud JP, Canet E, Delaperche MF, D’Allest AM (1987). Paradoxical inward rib cage motion during rapid eye movement sleep in infants and young children. J. Dev. Physiol..

[12] Devlieger H (1991). The diaphragm of the newborn infant: anatomical and ultrasonographic studies. J. Dev. Physiol..

[13] Vassilakopoulos, T. R. C. in *Clinical Respiratory Medicine* (ed. Albert, R. S. S. & Jett, J.) 135–146 (Mosby Elsevier, 2008).

[14] Sieck GC, Fournier M, Blanco CE (1991). Diaphragm muscle fatigue resistance during postnatal development. J. Appl. Physiol. (1985).

[15] Mortola JP (1987). Dynamics of breathing in newborn mammals. Physiol. Rev..

[16] Kosch PC, Hutchinson AA, Wozniak JA, Carlo WA, Stark AR (1988). Posterior cricoarytenoid and diaphragm activities during tidal breathing in neonates. J. Appl. Physiol. (1985).

[17] Davis GM, Coates AL, Papageorgiou A, Bureau MA (1988). Direct measurement of static chest wall compliance in animal and human neonates. J. Appl. Physiol. (1985).

[18] South M, Morley CJ, Hughes G (1987). Expiratory muscle activity in preterm babies. Arch. Dis. Child..

[19] Prechtl HF, van Eykern LA, O’Brien MJ (1977). Respiratory muscle EMG in newborns: a non-intrusive method. Early Hum. Dev..

[20] Muller N, Volgyesi G, Bryan MH, Bryan AC (1979). The consequences of diaphragmatic muscle fatigue in the newborn infant. J. Pediatr..

[21] Lopes JM, Muller NL, Bryan MH, Bryan AC (1981). Synergistic behavior of inspiratory muscles after diaphragmatic fatigue in the newborn. J. Appl. Physiol. Respir. Environ. Exerc. Physiol..

[22] Lopes J, Muller NL, Bryan MH, Bryan AC (1981). Importance of inspiratory muscle tone in maintenance of FRC in the newborn. J. Appl. Physiol. Respir. Environ. Exerc. Physiol..

[23] Carlo WA, Miller MJ, Martin RJ (1985). Differential response of respiratory muscles to airway occlusion in infants. J. Appl. Physiol. (1985).

[24] Curzi-Dascalova L (1982). Phase relationships between thoracic and abdominal respiratory movement during sleep in 31 - 38 weeks CA normal infants. Comparison with full-term (39 - 41 weeks) newborns. Neuropediatrics.

[25] Eichenwald EC, Ungarelli RA, Stark AR (1993). Hypercapnia increases expiratory braking in preterm infants. J. Appl. Physiol. (1985).

[26] Duara S, Silva Neto G, Claure N (1994). Role of respiratory muscles in upper airway narrowing induced by inspiratory loading in preterm infants. J. Appl. Physiol. (1985).

[27] Wulbrand H, Von Zezschwitz G, Bentele KH (1995). Submental and diaphragmatic muscle activity during and at resolution of mixed and obstructive apneas and cardiorespiratory arousal in preterm infants. Pediatr. Res..

[28] Hutten GJ (2008). Relative impact of respiratory muscle activity on tidal flow and end expiratory volume in healthy neonates. Pediatr. Pulmonol..

[29] Hutten GJ (2010). Respiratory muscle activity related to flow and lung volume in preterm infants compared with term infants. Pediatr. Res..

[30] Kosch PC, Stark AR (1984). Dynamic maintenance of end-expiratory lung volume in full-term infants. J. Appl. Physiol. Respir. Environ. Exerc. Physiol..

[31] Shardonofsky FR, Perez-Chada D, Carmuega E, Milic-Emili J (1989). Airway pressures during crying in healthy infants. Pediatr. Pulmonol..

[32] American Thoracic Society/European Respiratory Society. ATS/ERS Statement on respiratory muscle testing. *Am. J. Respir. Crit. Care Med*. **166**, 518–624 (2002).

[33] Scott CB (1983). Developmental pattern of maximal transdiaphragmatic pressure in infants during crying. Pediatr. Res..

[34] Dimitriou G, Greenoug A, Dyke H, Rafferty GF (2000). Maximal airway pressures during crying in healthy preterm and term neonates. Early Hum. Dev..

[35] Dimitriou G, Greenough A, Endo A, Cherian S, Rafferty GF (2002). Prediction of extubation failure in preterm infants. Arch. Dis. Child. Fetal Neonatal Ed..

[36] Currie A, Patel DS, Rafferty GF, Greenough A (2011). Prediction of extubation outcome in infants using the tension time index. Arch. Dis. Child. Fetal Neonatal Ed..

[37] Dimitriou G, Greenough A, Rafferty GF, Moxham J (2001). Effect of maturity on maximal transdiaphragmatic pressure in infants during crying. Am. J. Respir. Crit. Care Med..

[38] Decramer M, Scano G (1994). Assessment of respiratory muscle function. Eur. Respir. J..

[39] Gaultier C (1997). Tension-time index of inspiratory muscles in children. Pediatr. Pulmonol..

[40] Dimitriou G, Fouzas S, Vervenioti A, Tzifas S, Mantagos S (2011). Prediction of extubation outcome in preterm infants by composite extubation indices. Pediatr. Crit. Care Med..

[41] Mok Q, Ross-Russell R, Mulvey D, Green M, Shinebourne EA (1991). Phrenic nerve injury in infants and children undergoing cardiac surgery. Br. Heart J..

[42] Dimitriou G (2003). Diaphragmatic function in infants with surgically corrected anomalies. Pediatr. Res..

[43] Rafferty GF (2000). Assessment of neonatal diaphragm function using magnetic stimulation of the phrenic nerves. Am. J. Respir. Crit. Care Med..

[44] Dimitriou G, Greenough A, Moxham J, Rafferty GF (2003). Influence of maturation on infant diaphragm function assessed by magnetic stimulation of phrenic nerves. Pediatr. Pulmonol..

[45] Kassim Z, Jolley C, Moxham J, Greenough A, Rafferty GF (2011). Diaphragm electromyogram in infants with abdominal wall defects and congenital diaphragmatic hernia. Eur. Respir. J..

[46] Hammer J, Newth CJ (2009). Assessment of thoraco-abdominal asynchrony. Paediatr. Respir. Rev..

[47] Allen JL (1991). Interaction between chest wall motion and lung mechanics in normal infants and infants with bronchopulmonary dysplasia. Pediatr. Pulmonol..

[48] Locke R, Greenspan JS, Shaffer TH, Rubenstein SD, Wolfson MR (1991). Effect of nasal CPAP on thoracoabdominal motion in neonates with respiratory insufficiency. Pediatr. Pulmonol..

[49] Goldman MD (1993). Asynchronous chest wall movements during non-rapid eye movement and rapid eye movement sleep in children with bronchopulmonary dysplasia. Am. Rev. Respir. Dis..

[50] Praud JP (1991). Abdominal muscle activity during CO2 rebreathing in sleeping neonates. J. Appl. Physiol. (1985).

[51] Sidoroff, V. et al. The use of structured light plethysmography to assess the effect of feeding on tidal breathing patterns in newborns. *Arch. Dis. Child*. **98**, A73–A74 (2013).

[52] Dassios T (2015). Time constant of inspiratory muscle relaxation in cystic fibrosis. Pediatr. Res..

[53] Dassios T, Kaltsogianni O, Greenough A (2017). Relaxation rate of the respiratory muscles and prediction of extubation outcome in prematurely born infants. Neonatology.

[54] Miller LE, Stoller JZ, Fraga MV (2020). Point-of-care ultrasound in the neonatal ICU. Curr. Opin. Pediatr..

[55] Matamis D (2013). Sonographic evaluation of the diaphragm in critically ill patients. Technique and clinical applications. Intensive Care Med..

[56] Laing IA, Teele RL, Stark AR (1988). Diaphragmatic movement in newborn infants. J. Pediatr..

[57] Kocis KC (1997). Ultrasound evaluation of piglet diaphragm function before and after fatigue. J. Appl. Physiol. (1985).

[58] Alonso-Ojembarrena A (2020). Reproducibility and reference values of diaphragmatic shortening fraction for term and premature infants. Pediatr. Pulmonol..

[59] Donn, S. M. & Mammel, M. C. *Neonatal Pulmonary Graphics: A Clinical Pocket Atlas* (Springer, 2015).

[60] Hunt KA, Hunt I, Ali K, Dassios T, Greenough A (2020). Prediction of extubation success using the diaphragmatic electromyograph results in ventilated neonates. J. Perinat. Med..

[61] Sillos EM, Veber M, Schulman M, Krauss AN, Auld PA (1992). Characteristics associated with successful weaning in ventilator-dependent preterm infants. Am. J. Perinatol..

[62] Bhat P, Peacock JL, Rafferty GF, Hannam S, Greenough A (2016). Prediction of infant extubation outcomes using the tension-time index. Arch. Dis. Child. Fetal Neonatal Ed..

[63] Bahgat, E., El-Halaby, H., Abdelrahman, A., Nasef, N. & Abdel-Hady, H. Sonographic evaluation of diaphragmatic thickness and excursion as a predictor for successful extubation in mechanically ventilated preterm infants. *Eur. J. Pediatr*. **180**, 899–908 (2021). 10.1007/s00431-020-03805-2 PMC7520377 32986125

[64] Kraaijenga JV, de Waal CG, Hutten GJ, de Jongh FH, van Kaam AH (2017). Diaphragmatic activity during weaning from respiratory support in preterm infants. Arch. Dis. Child. Fetal Neonatal Ed..

[65] Jeffreys E, Hunt KA, Dassios T, Greenough A (2019). Diaphragm electromyography results at different high flow nasal cannula flow rates. Eur. J. Pediatr..

[66] Henderson-Smart DJ, Read DJ (1976). Depression of respiratory muscles and defective responses to nasal obstruction during active sleep in the newborn. Aust. Paediatr. J..

[67] Stark AR, Cohlan BA, Waggener TB, Frantz ID, Kosch PC (1987). Regulation of end-expiratory lung volume during sleep in premature infants. J. Appl. Physiol. (1985).

[68] Decramer M (1997). Hyperinflation and respiratory muscle interaction. Eur. Respir. J..

[69] Braun NM, Arora NS, Rochester DF (1982). Force-length relationship of the normal human diaphragm. J. Appl. Physiol. Respir. Environ. Exerc. Physiol..

[70] Roussos C (1985). Function and fatigue of respiratory muscles. Chest.

[71] Watchko JF, Standaert TA, Woodrum DE (1987). Diaphragmatic function during hypercapnia: neonatal and developmental aspects. J. Appl. Physiol. (1985).

[72] Watchko JF, LaFramboise WA, Standaert TA, Woodrum DE (1986). Diaphragmatic function during hypoxemia: neonatal and developmental aspects. J. Appl. Physiol. (1985).

[73] Rafferty GF, Lou Harris M, Polkey MI, Greenough A, Moxham J (1999). Effect of hypercapnia on maximal voluntary ventilation and diaphragm fatigue in normal humans. Am. J. Respir. Crit. Care Med..

[74] Wolfson MR, Greenspan JS, Deoras KS, Allen JL, Shaffer TH (1992). Effect of position on the mechanical interaction between the rib cage and abdomen in preterm infants. J. Appl. Physiol. (1985).

[75] Maynard V, Bignall S, Kitchen S (2000). Effect of positioning on respiratory synchrony in non-ventilated pre-term infants. Physiother. Res. Int..

[76] Dimitriou G (2002). Effect of posture on oxygenation and respiratory muscle strength in convalescent infants. Arch. Dis. Child. Fetal Neonatal Ed..

[77] Rao H (2009). Position and ventilatory response to added dead space in prematurely born infants. Pediatr. Pulmonol..

[78] Saiki, T. et al. Sleeping position and responses to a carbon dioxide challenge in convalescent prematurely born infants studied post-term. *Arch. Dis. Child. Fetal Neonatal Ed*. (2014). 10.1136/archdischild-2013-305586 24473750

[79] Dimitriou, G., Vervenioti, A., Papakonstantinou, D., Tzifas, S. & Mantagos, S. Effect of posture on work of breathing in preterm infants. *Early Hum. Dev.* **86**, S10 (2010).

[80] Williams AG (2000). The ACE gene and muscle performance. Nature.

[81] Myerson S (1999). Human angiotensin I-converting enzyme gene and endurance performance. J. Appl. Physiol. (1985).

[82] Dimitriou G (2010). Angiotensin-converting enzyme gene polymorphism and respiratory muscle function in infants. Pediatr. Pulmonol..

[83] Le Bourdelles G (1994). Effects of mechanical ventilation on diaphragmatic contractile properties in rats. Am. J. Respir. Crit. Care Med..

[84] Knisely AS, Leal SM, Singer DB (1988). Abnormalities of diaphragmatic muscle in neonates with ventilated lungs. J. Pediatr..

[85] Liang F (2019). Mechanical ventilation causes diaphragm dysfunction in newborn lambs. Crit. Care.

[86] de Waal CG, Hutten GJ, Kraaijenga JV, de Jongh FH, van Kaam AH (2017). Electrical activity of the diaphragm during nCPAP and high flow nasal cannula. Arch. Dis. Child. Fetal Neonatal Ed..

[87] Dassios T, Dixon P, Greenough A (2019). Ventilation efficiency and respiratory muscle function at different levels of CPAP in intubated prematurely born infants. Respir. Care.

[88] Jarreau PH (1996). Patient-triggered ventilation decreases the work of breathing in neonates. Am. J. Respir. Crit. Care Med..

[89] Manczur T, Greenough A, Rafferty GF (2000). Comparison of the pressure time product during synchronous intermittent mandatory ventilation and continuous positive airway pressure. Arch. Dis. Child..

[90] Patel DS, Sharma A, Prendergast M, Rafferty GF, Greenough A (2009). Work of breathing and different levels of volume-targeted ventilation. Pediatrics.

[91] Patel DS, Rafferty GF, Lee S, Hannam S, Greenough A (2009). Work of breathing during SIMV with and without pressure support. Arch. Dis. Child..

[92] Vervenioti A, Fouzas S, Tzifas S, Karatza AA, Dimitriou G (2020). Work of breathing in mechanically ventilated preterm neonates. Pediatr. Crit. Care Med..

[93] Eichenwald EC (1992). Developmental changes in sequential activation of laryngeal abductor muscle and diaphragm in infants. J. Appl. Physiol. (1985).

[94] Kassim Z, Greenough A, Rafferty GF (2009). Effect of caffeine on respiratory muscle strength and lung function in prematurely born, ventilated infants. Eur. J. Pediatr..

[95] Kraaijenga JV, Hutten GJ, de Jongh FH, van Kaam AH (2015). The effect of caffeine on diaphragmatic activity and tidal volume in preterm infants. J. Pediatr..

[96] Williams EE (2020). Electrical activity of the diaphragm following a loading dose of caffeine citrate in ventilated preterm infants. Pediatr. Res..

[97] Benameur M, Goldman MD, Ecoffey C, Gaultier C (1993). Ventilation and thoracoabdominal asynchrony during halothane anesthesia in infants. J. Appl. Physiol. (1985).

[98] Trang TT, Viires N, Aubier M (1992). Effect of steroids on diaphragm of newborn, weanling adolescent, and adult rats. Am. Rev. Respir. Dis..

[99] Nava S (1996). Effects of acute steroid administration on ventilatory and peripheral muscles in rats. Am. J. Respir. Crit. Care Med..

[100] Song Y (2014). Effect of maternal steroid on developing diaphragm integrity. PLoS ONE.

[101] Froese AB, Bryan AC (1974). Effects of anesthesia and paralysis on diaphragmatic mechanics in man. Anesthesiology.

[102] Song Y (2018). Vitamin A protects the preterm lamb diaphragm against adverse effects of mechanical ventilation. Front. Physiol..

[103] Khirani S (2018). Diaphragmatic function in infants and children with congenital diaphragmatic hernia: a cross-sectional study. Eur. J. Cardiothorac. Surg..

[104] Dassios T, Kaltsogianni O, Dixon P, Greenough A (2018). Effect of maturity and infection on the rate of relaxation of the respiratory muscles in ventilated, newborn infants. Acta Paediatr..

[105] Song Y (2013). In utero LPS exposure impairs preterm diaphragm contractility. Am. J. Respir. Cell Mol. Biol..

[106] Stein H, Firestone K (2014). Application of neurally adjusted ventilatory assist in neonates. Semin. Fetal Neonatal Med..

[107] Shetty S, Hunt K, Peacock J, Ali K, Greenough A (2017). Crossover study of assist control ventilation and neurally adjusted ventilatory assist. Eur. J. Pediatr..

[108] Hunt KA, Dassios T, Greenough A (2020). Proportional assist ventilation (PAV) versus neurally adjusted ventilator assist (NAVA): effect on oxygenation in infants with evolving or established bronchopulmonary dysplasia. Eur. J. Pediatr..

[109] Kallio M (2019). NIV NAVA versus nasal CPAP in premature infants: a randomized clinical trial. Neonatology.

[110] Rochon ME (2020). Continuous neurally adjusted ventilation: a feasibility study in preterm infants. Arch. Dis. Child. Fetal Neonatal Ed..

[111] de Waal CG, Kraaijenga JV, Hutten GJ, de Jongh FH, van Kaam AH (2017). Breath detection by transcutaneous electromyography of the diaphragm and the Graseby capsule in preterm infants. Pediatr. Pulmonol..

[112] Brunherotti MA, Bezerra PP, Bachur CK, Jacometti CR (2012). Inspiratory muscle training in a newborn with anoxia who was chronically ventilated. Phys. Ther..

[113] Smith BK, Bleiweis MS, Neel CR, Martin AD (2013). Inspiratory muscle strength training in infants with congenital heart disease and prolonged mechanical ventilation: a case report. Phys. Ther..

[114] Fleming PJ, Muller NL, Bryan MH, Bryan AC (1979). The effects of abdominal loading on rib cage distortion in premature infants. Pediatrics.

[115] Nichols DG (1991). Respiratory muscle performance in infants and children. J. Pediatr..

